# Large is different: Nonmonotonic behavior of elastic range scaling in polymeric turbulence at large Reynolds and Deborah numbers

**DOI:** 10.1126/sciadv.add3831

**Published:** 2023-03-15

**Authors:** Marco E. Rosti, Prasad Perlekar, Dhrubaditya Mitra

**Affiliations:** ^1^ Complex Fluids and Flows Unit, Okinawa Institute of Science and Technology Graduate University, 1919-1 Tancha, Onna-son, Okinawa 904-0495, Japan.; ^2^ TIFR Centre for Interdisciplinary Sciences, Tata Institute of Fundamental Research, Gopanpally, Hyderabad 500046, India.; ^3^ Nordita, KTH Royal Institute of Technology and Stockholm University, Roslagstullsbacken 23, 10691 Stockholm, Sweden.

## Abstract

We use direct numerical simulations to study homogeneous and isotropic turbulent flows of dilute polymer solutions at high Reynolds and Deborah numbers. We find that for small wave numbers *k*, the kinetic energy spectrum shows Kolmogorov-like behavior that crosses over at a larger *k* to a novel, elastic scaling regime, *E*(*k*) ∼ *k*^−ξ^, with ξ ≈ 2.3. We study the contribution of the polymers to the flux of kinetic energy through scales and find that it can be decomposed into two parts: one increase in effective viscous dissipation and a purely elastic contribution that dominates over the nonlinear flux in the range of *k* over which the elastic scaling is observed. The multiscale balance between the two fluxes determines the crossover wave number that depends nonmonotically on the Deborah number. Consistently, structure functions also show two scaling ranges, with intermittency present in both of them in equal measure.

## Teaser

An elastic range in polymeric turbulence at large Reynolds numbers is uncovered and is non-monotonic with the Deborah number.

## INTRODUCTION

Since the discovery of turbulent drag reduction by Toms (*1*), turbulent flows with small amount of long-chained polymers have remained an exciting field of research. In addition to polymer concentration, two dimensionless numbers, the Reynolds number and the Deborah number, are necessary to describe such a turbulent flow. The former estimates the importance of the inertial term in the Navier-Stokes equation compared to the viscous term, and the latter is the ratio of the characteristic time scale of the polymers over the typical time scale of the large-scale eddies in the turbulent flow. The turbulent drag reduction appears at both large Reynolds and Deborah numbers. Evidently, it is not possible to study drag reduction in homogeneous and isotropic turbulent flows; nevertheless, such flows are studied, since the pioneering work by Tabor and De Gennes (*2*), in search of deeper insights.

The elementary effect of the addition of polymers to a fluid is an increase in the effective viscosity of the solution (*3*, *4*). Nevertheless, there can be net reduction of the dissipation of kinetic energy (*5*–*11*) because the presence of polymers changes the turbulent cascade qualitatively. Past theoretical (*2*, *4*, *12*–*14*), numerical (*8*, *10*, *11*, *15*–*26*), and experimental (*5*, *27*–*33*) efforts have gone into elucidating the nature of the turbulent energy cascade in the presence of polymers. It is now reasonably well established (*8*, *10*) that for large enough scale separation between the energy injection scale, *L*_inj_, and the Kolmogorov scale, *L*_K_, there exists an intermediate scale *L*_p_ such that for scales *L*_p_ < *r* < *L*_inj_ the energy cascade is practically the same as that of a Newtonian flow, with the second-order structure function *S*_2_(*r*) ∼ *r*^2/3^ and the shell-integrated energy spectrum being *E*(*k*) ∼ *k*^−5/3^. For scales *r* in the range *L*_K_ < *r* < *L*_p_, energy is transferred from the fluid to the polymers, and the kinetic energy spectrum is steeper than the Kolmogorov spectrum or in other words the second-order structure function increases faster with *r* than *r*^2/3^. Using the concept of scale-dependent Reynolds number (*34*), we may identify the flow at scale *r* < *L*_p_ (also valid for *r* < *L*_K_) with elastic turbulence—random viscoelastic smooth flows at very small Reynolds number. The spectrum for elastic turbulence is expected to be *E*(*k*) ∼ *k*^−ξ^ with ξ > 3 (*14*, *35*, *36*). Is there a previously unidentified scaling range for *r* < *L*_p_ over which *S*_2_(*r*) ∼ *r*^ζ_2_^ with 2/3 < ζ_2_ < 2? This question could not be probed with the low-Reynolds and low-Deborah simulations quoted above. Recent experiments (*33*) had tentatively suggested that a new scaling range indeed appears, although the evidence is not yet unequivocal. Experiments (*33*, *37*) also showed that, contrary to Lumley’s arguments (*38*), the scale *L*_p_ does depends on the concentration of polymers.

Here, we present evidence, from the highest-resolution three-dimensional simulations of polymeric fluids, that indeed there is a range of scales *r* over which the structure function *S*_2_(*r*) seems to show scaling consistent with recent experimental results (*33*). We also show that the new scaling is a purely elastic effect and that this elastic behavior is nonmonotonic in the Deborah number.

## RESULTS

### Governing equations

We use direct numerical simulations to study three-dimensional homogeneous isotropic turbulence with polymers (*3*, *39*–*43*). These are represented by a second-rank tensor, C with components *C*_αβ_, which emerges as the thermal average of the tensor product of the polymer end-to-end distance with itself. The polymer molecules are assumed to have a single relaxation time τ_p_. The dynamical equations are

$$\rho_{f}\left(\frac{\partial u_{\alpha }}{\partial t}+\frac{\partial u_{\alpha }u_{\beta }}{\partial x_{\beta }}\right)=-\frac{\partial p}{\partial x_{\alpha }}+\frac{\partial }{\partial x_{\beta }}\left(2\mu_{f}S_{\alpha \beta }+\frac{\mu_{p}}{\tau_{p}}fC_{\alpha \beta }\right)+F_{\alpha }$$ (1A)

$$\frac{\partial C_{\alpha \beta }}{\partial t}+u_{\gamma }\frac{\partial C_{\alpha \beta }}{\partial x_{\gamma }}=C_{\alpha \gamma }\frac{\partial u_{\beta }}{\partial x_{\gamma }}+C_{\gamma \beta }\frac{\partial u_{\gamma }}{\partial x_{\alpha }}-\frac{fC_{\alpha \beta }-\delta_{\alpha \beta }}{\tau_{p}}$$ (1B)

$$\frac{\partial u_{\alpha }}{\partial x_{\alpha }}=0$$ (1C)

Here *u* is the velocity, ρ_f_ = 1 and μ_f_ are the density and dynamic viscosity of the fluid, *p* is the pressure, μ_p_ is the polymer viscosity, and S is the rate-of-strain tensor with components *S*_αβ_ defined as *S*_αβ_ = (*∂u*_α_/*∂x*_β_ + *∂u*_β_/*∂x*_α_)/2. The function *f* is equal to unity (*f* = 1) in the purely elastic Oldroyd-B model and to *f* = (ℒ^2^ − 3)/(ℒ^2^ − *C*_γγ_) in the Finitely Extensible Nonlinear Elastic - Peterlin (FENE-P) model (where ℒ is the maximum allowed extension of the polymers) exhibiting both shear thinning and elasticity. The polymer time scale is the relaxation time τ_p_ and its concentration is related to the value of 1 + μ_p_/μ_f_; the value chosen in this work corresponds, roughly, to 100 ppm for polyethylene oxide (*44*). Note that we work in the dilute limit where polymer concentration is assumed to be homogeneous. Turbulence is sustained by the external force in the momentum equation, *F*; we use the spectral scheme from (*45*) to randomly inject energy to the low–wave number shells with *k*_inj_ = (1 ≤ *k* ≤ 2). Note that the scaling behavior in wave numbers much larger than *k*_inj_ is independent of the choice of *k*_inj_. In the statistically stationary state of turbulence, the injected energy is dissipated by both the fluid (ε_f_) and the polymers (ε_p_), thus ε_inj_ = ε_f_ + ε_p_, where

$$\varepsilon_{f}=\frac{2\mu_{f}}{\rho_{f}}\langle S_{\alpha \beta }S_{\alpha \beta }\rangle ,\varepsilon_{p}=\frac{\mu_{p}}{2\rho_{f}\tau_{p}^{2}}\langle f(fC_{\mu \mu }-3)\rangle$$ (2)

To compare, we also solve for the Navier-Stokes equations without any polymer additive—we call this the Newtonian simulation.

### Theoretical background

Let us briefly recall essential features of fluid turbulence without polymeric additives (*46*). The flow is determined by one dimensionless number, Re = u_rms_/(*k*_inj_ν_f_), where u_rms_ is the root mean square velocity and ν_f_ = μ_f_/ρ_f_ is the kinematic viscosity of the fluid. Turbulent flows have a range of length scales and corresponding time scales. The statistical properties of such flows are characterized by the scaling exponents, ζ_q_ of the qth order longitudinal structure functions, *S_q_*, defined by

$$S_{q}(\ell )=\langle \delta u(\ell {)}^{q}∼\ell^{\zeta q}\rangle \;\mathrm{where}$$ (3A)

$$\delta u(\ell )\equiv [u(x+\ell )-u(x)]\cdot \left(\frac{\ell }{\ell }\right)$$ (3B)

Here, ⟨·⟩ denotes averaging over the statistically stationary state of turbulence. The *q*th order structure function is the *q*th order moment of the probability distribution function (PDF) of velocity difference across a length scale ℓ. The scaling behavior of the structure function, Eq. 3A, holds for η ≪ ℓ ≪ *L* where $\eta \equiv {\left(\nu_{f}^{3}/\varepsilon_{\mathrm{inj}}\right)}^{1/4}$ is called the viscous scale and *L* is called the integral scale. In practice, *L* = 2π/*k*_L_ is of the same order of *L*_inj_ = 2π/*k*_inj_, and we will use them interchangeably.

The shell-integrated energy spectrum in Fourier space

$$E(k)\equiv \int_{|m|=k}d^{3}m\langle \hat{u}(m)\hat{u}(-m)\rangle$$ (4)

where $\hat{u}(m)$ is the Fourier transform of the velocity field *u*(*x*) and is itself the Fourier transform of the second-order structure function *S*_2_(ℓ). The theory of Kolmogorov gives ζ_q_ = *q*/3 and consequently *E*(*k*) ∼ *k*^−5/3^, when *k* lies within the inertial range, *k*_inj_ ≪ *k* ≪ *k*_η_, with *k*_η_ ∼ 1/η. The turbulent velocity fluctuations are non-Gaussian in two ways. First, the odd-order structure functions are nonzero, particularly the third-order structure function satisfies the most celebrated exact relation in turbulence, i.e., the four-fifth law, *S*_3_(ℓ) = −(4/5)ε_inj_ℓ—this result is the cornerstone of Kolmogorv’s theory of turbulence. Second, the scaling exponents ζ_q_ are a nonlinear convex function of q, a phenomena called intermittency.

In the presence of polymers, we, in addition, consider

$$E_{p}(k)\equiv \left(\frac{\mu_{p}}{\rho_{f}\tau_{p}}\right)\int_{|m|=k}d^{3}m\langle {\hat{B}}_{\gamma \beta }(m){\hat{B}}_{\beta \gamma }(-m)\rangle$$ (5)

where the matrix ℬ with components, *B*_αγ_, is the (unique) positive symmetric square root of the matrix C, i.e., *C*_αβ_ = *B*_αγ_*B*_γβ_ (*24*, *47*). For the Oldroyd-B model, the total energy in the polymeric mode is given by

$$E_{p}\equiv \frac{1}{2}\int dkE_{p}(k)=\frac{\mu_{p}}{2\rho_{f}\tau_{p}}\langle C_{\mu \mu }\rangle$$ (6)

The presence of polymers introduces also an additional dimensionless number which is the ratio of the polymeric time scale τ_p_ over a characteristic time scale of the flow. As the turbulent flow has many time scales, it is common to define the Deborah number De ≡ τ_p_/τ_L_, where τ_L_ = *L*/u_rms_ is the large-eddy turnover time, and the Weissenberg number Wi ≡ τ_p_/τ_η_, where τ_η_ = η^2^/ν (*48*).

**Fig. 1.**
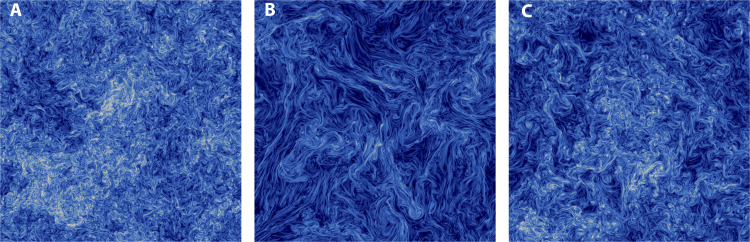
Instantaneous snapshots of the turbulent flows. (**A**) Newtonian and viscoelastic fluid with (**B**) *De* ≈ 1 and (**C**) De ≈ 25, at a nominal microscale Reynolds number Re_λ_ ≈ 400 for the Newtonian case (A, Re_λ_ = 390; B, Re_λ_ ≈ 740; C, Re_λ_ ≈ 447). The color contour shows the magnitude of the vorticity field, with the color scale going from 0 (blue) to the maximum (white). The figures are two-dimensional cuts of the three-dimensional periodic cube passing through the middle of the domain.

In Fig. 1, we show typical pseudocolor plots of vorticity from Newtonian and viscoelastic simulations. The flow is qualitatively strongly affected by the presence of polymers, and small-scale vorticity structures are smoothened by the presence of the polymers, as can be seen by comparing Fig. 1 (A and B) [see also (*8*, *10*, *17*)]. Unexpectedly, as the Deborah number is increased beyond unity, this qualitative trend is reversed, compare Fig. 1 (B and C). In Fig. 1C, small-scale structures in vorticity reappears, but at the same time, we still find elongated structures although their length scales are smaller than their counterparts in Fig. 1B.

### Kinetic and polymer energy spectra

**Fig. 2.**
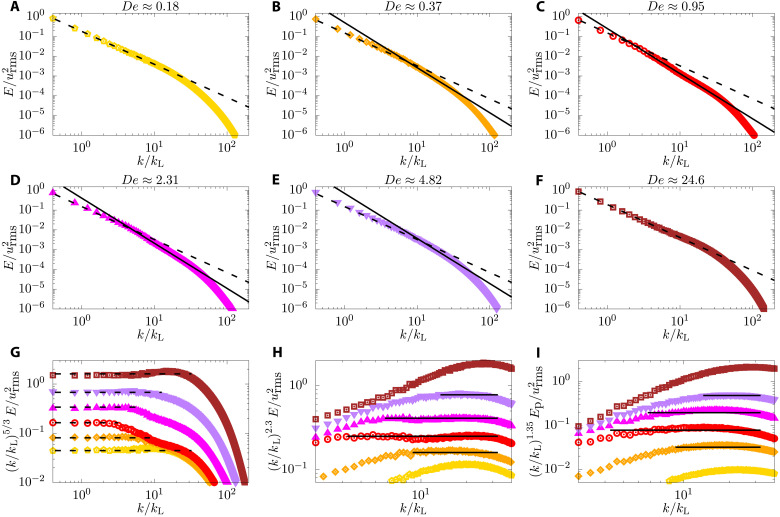
Kinetic and polymer energy spectra. (**A** to **F**) Energy spectra for different Deborah numbers showing how the elastic range changes with De.  (**G** and **H**) Compensated kinetic energy spectra showing the emergence of two scaling regions, Kolmogorov (−5/3) and elastic (−2.3) scaling, respectively. In all the previous panels, the dashed and solid lines represent the −5/3 and −2.3 scalings. (**I**) Compensated polymer energy spectra for different Deborah numbers. The solid line represent the scaling *k*^−ψ^ with ψ ≈ 1.35. The scaling laws in (G) to (I) extend over the scales found from the crossovers extracted from Fig. 3. The abscissa are normalized with the integral length scale wave number *k*_L_.

In Fig. 2, we plot the turbulent kinetic energy *E*(*k*) for several values of Deborah number De.  For the small Deborah numbers, e.g., De ≈ 0.18, we observe, practically, the same behavior as Kolmogorov turbulence, with *E*(*k*) ∼ *k*^−5/3^ for the inertial range. As the Deborah number increases the range over which the Kolmogorov scaling is valid shrinks to smaller *k*, and at intermediate *k*, a new range over which *E*(*k*) ∼ *k*^−ξ^ with ξ ≈ 2.3 emerges. We call this new scaling range the elastic range. The spectra, in general, has three characteristic length scales (or equivalently wave numbers). The largest is the one where energy is injected by stirring, the integral scale, *L* ≈ *L*_inj_. Next is the scale at which the Kolmogorov scaling crosses over to elastic scaling, *L*_p_ (corresponding wave number *k*_p_ = 2π/*L*_p_). Last is the scale at which elastic scaling crosses over to the dissipative range, which we call the dissipative scale η (corresponding wave number *k*_η_ = 1/η). The Kolmogorov scaling is observed over the range *k*_inj_ < *k* < *k_p_* and the elastic range over the wave number range *k_p_* < *k* < *k*_η_. The elastic range spans over the maximum range of wave number for De ≈ 1; as De is increased further, the elastic range begins to shrink and the Kolmogorov range begins to grow again. Eventually, the elastic range practically disappears at De ≈ 5, the classical Kolmogorov range is restored. This remarkable behavior is better elucidated by plotting the two compensated spectra *k*^5/3^*E*(*k*) and *k^ξ^E*(*k*) in Fig. 2 (G and H), respectively. In other words, our results clearly show that the wave number *k*_p_ depends nonmonotonically on the Deborah number, being maximum for De ≈ 1. The nonmonotonic behavior of the polymeric flow for De ≈ 1 can be also qualitatively appreciated by observing Fig. 1.

Although the steepening of the spectra beyond a certain wave number *k* = *k*_p_ have been observed before in both direct numerical simulations (*8*, *10*, *17*, *18*, *26*) and experiments (*32*), this was mostly confined to the dissipation range due to the small separation of scales related to the Reynolds number considered; only recent experiments (*33*) demonstrated for the first time the emergence of this elastic scaling. Zhang *et al.* (*33*) also found that *L*_p_ increases with the polymer concentration, but they did not investigate how it behaves as a function of the Deborah number. Also, we find both Kolmogorov and elastic scaling simultaneously valid for different ranges of wave numbers by virtue of running the largest simulation of polymeric turbulence so far (see fig. S1), and we also uncover the nonmonotonic behavior of *L*_p_ as a function of the Deborah number. We have also confirmed that these results are robust with respect to change in spatial and temporal resolutions (see fig. S2).

The previous results have been obtained for the purely elastic Oldroyd-B model. Before we explore further the elastic scaling, it is worth mentioning that we performed the simulation for De ≈ 0.95 with two additional models of polymeric fluids—the inelastic, shear thinning Carreau-Yasuda model and the FENE-P model, which models both the elastic and shear thinning behavior of polymeric fluids. We find that the new scaling at intermediate scales is a purely elastic effect, which completely disappear in the absence of elasticity, while it is reduced when shear thinning is present together with elasticity (see fig. S3). We have also observed that if the parameter ℒ (the maximum possible extension of the polymers) of the FENE-P model is varied within a reasonable range, the elastic scaling remains practically unchanged. For too small a value of ℒ the elastic scaling range can disappear (see fig. S3B).

### Scale-by-scale energy budget

In turbulence, to understand the energy spectra, we have to study the flux of energy through scales (*46*, *49*, *50*). For polymeric turbulence, the flux in Fourier space have been studied before in (*25*, *26*) and their real space analog in (*17*).

To obtain the flux of kinetic energy in Fourier space, transform Eq. 1A to Fourier space, multiply by $\hat{u}(-k)$, integrate over the solid angle *d*Ω and over *k* from 0 to *K*, and average over the statistically stationary state of turbulence to obtain

$$\epsilon_{\mathrm{inj}}=\epsilon_{f}+\epsilon_{p}={\text{Π}}_{f}(K)+D_{f}(K)+P(K)+F_{\mathrm{inj}}(K)$$ (7)

where Π_f_, D_f_, P, and ℱ_inj_ are the contributions from the nonlinear term, the viscous term, the polymeric stress, and the external force in Eq. 1A (see the Supplementary Materials for a full derivation). The first equality of Eq. 7 follows from statistical stationarity. For *K* ≫ *k*_inj_, the external force is zero. In the absence of polymers, P = 0, and, since in the inertial range the dissipative contribution D_f_ is negligible, Π_f_(*K* ≫ *k*_inj_) = ε_inj_ is a constant. The Kolmogorov four-fifth law follows from this statement (*46*). In addition, if we assume that scaling, we obtain *E*(*k*) ∼ *k*^−5/3^.

**Fig. 3.**
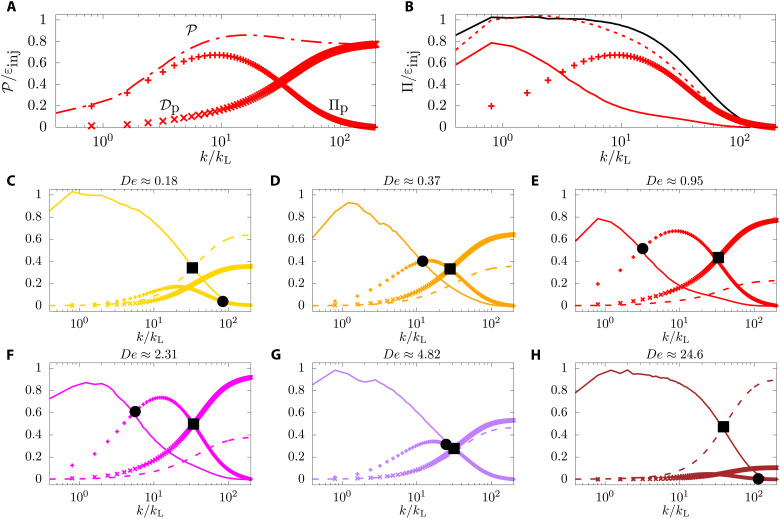
Scale-by-scale energy budget. (**A**) The polymer contribution to the spectral energy balance, P, (dash-dotted line) is decomposed into a (×) pure polymer dissipation term, D_p_, and a (+) pure polymer energy flux, Π_p_, see Eq. 8A. (**B**) The sum of the (solid line) nonlinear energy flux Π_f_ and of the (+) polymer flux Π_p_ provides a (dotted line) total flux Π = Π_f_ + Π_p_ extending over a range comparable to the Newtonian case (black line). (**C** to **H**) The panels show the nonlinear energy flux Π_f_ (solid line), fluid dissipation D_f_ (dashed line), polymer flux, Π_p_ (+), and polymer dissipation D_p_ (×) for different Deborah numbers. The filled circles represent *k*_p_, and the filled squares represent *k*_η_, used in Fig. 2 (G to I) as the extension of the scaling laws. As De approaches unity, the polymer flux and dissipation grow, while they decrease for larger values. The abscissa are normalized with the integral length scale wave number *k*_L_. The color scheme is the same used in Fig. 2.

The novel physics of this problem is elucidated by studying the contribution from the polymers, P. In Fig. 3A, we show a representative plot of P(*K*) as a function of *K*, plotted as a dashed dotted line. It is well established (*3*, *4*, *42*) that one of the effects of addition of polymers to flows is the increase of dissipation, but P(*K*) is not a purely dissipative term, as shown by its nonmonotonicty with *K*. This feature has been modeled before by a wave number–dependent effective viscosity (*8*). Here, we try a different approach. We separate the part of P(*K*) that is purely dissipative, D_p_(*K*), such that at large *K* such a term should have the same asymptotic dependence on *K* as D_f_(*K*). We further demand that as *K* → ∞, D_p_(*K*) → ε_p_. Hence we obtain

$$P(K)={\text{Π}}_{p}(K)+D_{p}(K),\;\mathrm{where}$$ (8A)

$$D_{p}(K)\equiv \frac{\epsilon_{p}}{\epsilon_{f}}D_{f}(K).$$ (8B)

We plot Π_p_ and D_p_ individually in Fig. 3A. Π_p_ has the same qualitative behavior as Π_f_, the nonlinear flux. In Fig. 3B, we plot both Π_p_ and Π_f_ denoted by the symbol + and a continuous line, respectively. We find that for small *K*, Π_f_ is dominant and Π_p_ is negligible. At a certain scale k_*_, the two fluxes cross each other. Beyond k_*_, Π_p_ is the dominant partner and Π_f_ is negligible. At very large *K*, well within the dissipation range, both Π_f_ and Π_p_ go to zero. The sum of these two fluxes Π ≡ Π_p_ + Π_f_ is practically a constant for all *K* ≪ *k*_η_. In the same figure, Fig. 3B, we also plot, as a black line the contribution to the flux from the nonlinear term for a simulation with no polymers. The flux that is carried by the nonlinear term in the absence of polymers is carried by both Π_f_ and Π_p_ in the presence of polymers: At small *K*, the flux is carried mainly by Π_f_ and at large *K* the flux is carried mainly by Π_p_. The crossover between these two fluxes happens at k_*_, which we identify with *k*_p_. The fluxes clearly illustrate and substantiate what we already observed in the energy spectra: For *k* < *k*_p_, the turbulence is Kolmogorov-like, whereas for *k*_p_ < *k* < *k*_η_, the polymeric flux Π_p_ dominates and is approximately a constant. We define the range of Fourier modes, *k*_p_ < *k* < *k*_η_ as the elastic range with *k*_p_ precisely defined as Π_f_(*k*_p_) = Π_p_(*k*_p_).

We emphasize that the decomposition in Eqs. 8A and 8B is justified on the following grounds: First, by construction, D_p_(*K*) is always positive and monotonically increasing with *K*; second, it has the same asymptotic dependence on *K* as D_f_. While a direct consequence of this decomposition is that Π_p_ → 0 as *K* → ∞, this does not automatically guarantee that net flux Π(*K*) = Π_p_(*K*) + Π_f_(*K*) is almost a constant over a large range of scales at all De.  Our numerical results thus provide an additional post-facto justification of the decomposition of P. Also, we have checked that other reasonable choices for D_p_ do not change the results qualitatively.

For a moment, consider again turbulence without polymers. Assume that within the inertial range, in real space, the velocity shows scaling behavior with an exponent *h* such that, if we scale length by a factor of *b*, *x* → *bx*, then velocity scales as *u* → *b^h^u*. In the inertial range, the flux equation, Eq. 7, implies that the contribution to the flux from the nonlinear term is constant. Applying simple power counting to the contribution to the flux from the nonlinear term, we obtain 3*h* − 1 = 0, which implies the standard result from Kolmogorov theory *h* = 1/3. Let us now apply the same scaling argument to the elastic range (*17*). As we scale *x* → *bx*, we expect *u* → *b^h^u* and *C* → *b^g^C* with two distinct exponents *h* and *g*, respectively. As the flux Π_p_ is approximately constant in the elastic range, we obtain *h* − 1 + *g* = 0. By Fourier transform, it is straightforward to show that, if the velocity in real space scales with an exponent *h*, then the scaling exponent for the energy, *E*(*k*) ∼ *k*^−ξ^, with ξ = 2*h* + 1. Together, the two relations imply that the scaling exponent for the shell-integrated polymer energy is *E*_p_(*k*) ∼ *k*^−ψ^, with ψ = *g* + 1 = 2 − (ξ − 1)/2 ≈ 1.35. In Fig. 2I, we plot the compensated shell-integrated polymer spectra from our simulations; a scaling exponent of ψ ≈ 1.35 is indeed consistent with our results, independently corroborating the view of a polymer flux.

Next, we show how the flux balance depends on the Deborah number in Fig. 3 (C to H). We mark two Fourier modes in these plots, one is *k*_p_, marked by a black circle, the wave number at which Π_f_ stops being the dominant contribution, and the other is the wave number at which the dissipation (D_f_ or D_p_) becomes the dominant term of the balance, which is a reasonable estimate of *k*_η_, marked by a black square. For small De (Fig. 3C), *k*_p_ > *k*_η_; in other words, the elastic range is nonexistent, masked by the viscous range. As De increases (Fig. 3, D and E), *k*_p_ < *k*_η_ and the elastic range is clearly visible, with *k*_p_ reducing with De. As De increases beyond unity, *k*_p_ starts increasing again (Fig. 3F) and becomes almost equal to *k*_η_ in Fig. 3G. For even larger De, the elastic range disappears again. The values of *k*_p_ and *k*_η_ obtained from Fig. 3 are used in Fig. 2 (G to I) as the extension of the scaling laws; the agreement between the two is an independent verification of the validity of Eqs. 8A and 8B.

**Fig. 4.**
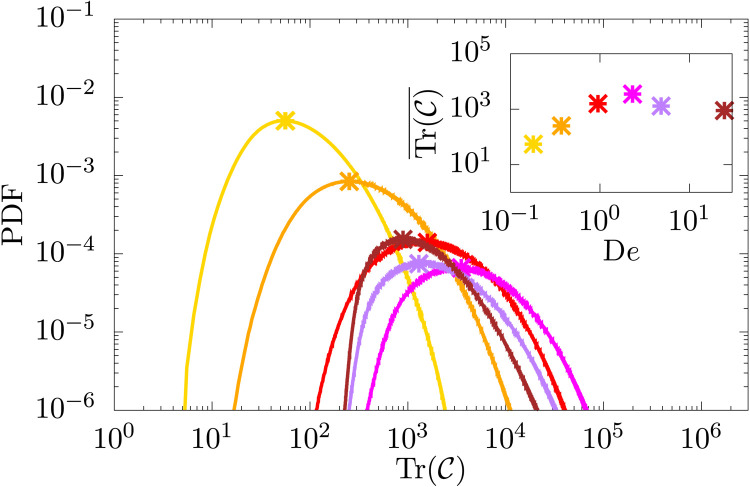
Polymer extension. PDF of the squared extension of the polymer for different Deborah numbers, measured in terms of Tr(C). The yellow, orange, red, magenta, purple, and brown colors are used for increasing values of Deborah numbers, and the color scheme is the same used in Fig. 2. The mean polymer extension (marked with a cross) is a nonmonotonic function of the Deborah number, as shown in the inset of the figure.

Last, this nonmonotonic behavior of the scale *k*_p_ is also reflected in the PDF of the squared extension of the polymers, Tr(C), shown in Fig. 4 for the Oldroyd-B model. For small Deborah numbers, the PDF has a peak somewhat higher than 3, i.e., some polymers are already not in a coiled state. This is expected because the stretching of polymers is determined by the small-scale strain rate (*51*–*53*) that is best captured by the Weissenberg number, which is about 16 for the smallest Deborah number we used. As De is increased, the peak of the PDF moves to higher and higher values, which is also what is expected. Unexpectedly, for De > 1, the peak moves back to smaller values. This is an effect that cannot be captured from a passive polymer theory (*48*)—the feedback from the polymer to the flow changes the strain rate such that, in turn, the stretching of the polymers is reversed at Deborah number greater than unity. Note that here we show results from the Oldroyd-B model where there is no constraint on the maximum stretching of polymers; however, this nonmonotonic behavior is not unique to the Oldroyd-B model, and we observe it also with the FENE-P model (see fig. S4B). Furthermore, since the polymer extension does not continuously increase with the Deborah number, the solution remains effectively dilute also at these high values of Weissenberg numbers, without invalidating the dilute hypothesis of the models used.

To summarize, we have, so far, presented evidence from the largest-resolution direct numerical simulations of polymeric turbulence that if the Deborah number lies in the right range, *k*_p_ < *k* < *k*_η_, an elastic range with constant polymeric flux Π_p_ emerges in which *E*(*k*) ∼ *k*^−ξ^, with ξ ≈ 2.3 and *E*_p_(*k*) ∼ *k*^−ψ^ with ψ ≈ 1.35. Crucially, the scale *k*_p_ behaves nonmonotonically as a function of De and can be precisely determined as the crossover scale between Π_f_ and Π_p_.

### Structure function and intermittency

In the absence of polymers, the scaling exponents of the structure function ζ_q_ are a nonlinear function of *q*—a phenomena known as intermittency (*46*), which can be parametrized by corrections to the Kolmogorov scaling

$$\zeta_{q}=q/3+\delta_{q}$$ (9)

**Fig. 5.**
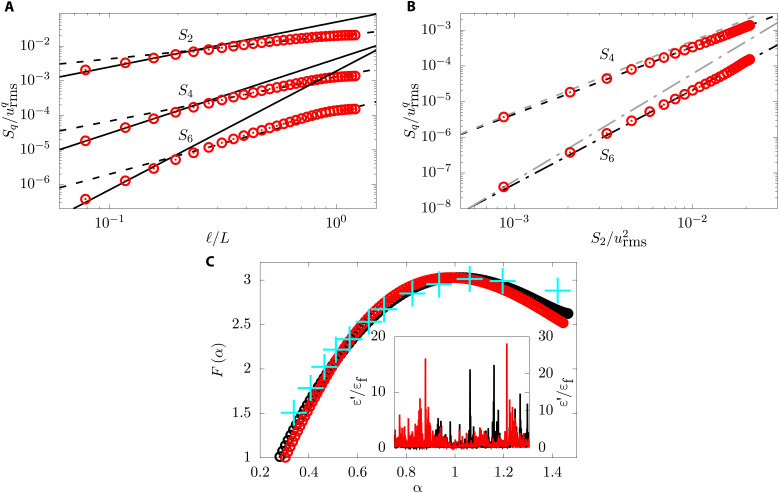
Intermittency. (**A**) Structure function *S_q_* for *q* = 2, 4, and 6 from top to bottom for the viscoelastic case at De ≈ 0.9. The solid and dashed lines represent the expected scalings in the polymer and inertial range of scales, corrected by the intermittency correction. (**B**) Same structure functions of (A), plotted in their extended self-similarity form (*55*). The black and gray lines represent the expected scaling with and without intermittency corrections. The polymer and inertial range of scales follow the same line, indicating that the intermittency in the two ranges is the same. (**C**) Multifractal spectrum of the fluid dissipation. The cyan symbols represent experimental data taken from (*56*). The inset shows typical signals for the fluid dissipation, normalized by its mean value. In the two figures, the Newtonian and viscoelastic cases are shown in black and red, respectively.

The best estimates (*54*) of δ_q_ are δ_2_ ≈ 0.04, δ_4_ ≈ − 0.05, and δ_6_ ≈ − 0.23, whereas δ_3_ = 0 is due to Kolmogorov’s four-fifth law. We now explore what happens to intermittency on the addition of polymers. In Fig. 5A, we plot the structure function for *q* = 2,4, and 6 for De ≈ 0.9—the case for which we have the largest elastic range. The second-order structure function (Eq. 3A) with *q* = 2 is the Fourier transform of the energy spectrum *E(k)*. Hence, if *E*(*k*) ∼ *k*^−ξ^, then *S*_2_(ℓ) ∼ ℓ^ζ_2_^, with ζ_2_ = ξ − 1 ≈ 1.3, which is what we obtain. On the other hand, the scalings for *q* = 4 and *q* = 6 are different from 2ζ_2_ and 3ζ_2_; this becomes obvious when we plot in Fig. 5B *S*_4_ and *S*_6_ as a function of *S*_2_ (*55*). In these plots, the elastic range and the inertial range seem to merge into one scaling range, suggesting that the intermittency correction for *q* = 4 and *q* = 6 is the same in both the elastic and the inertial ranges. Our results on intermittency discussed so far agree with the experimental results obtained in (*33*). Thus, we must conclude that the effect of the polymers is to change the dominant exponent *q*/3 but not the intermittency correction δ*_q_*. The dominant exponent is determined by the scaling of the mean value of the energy flux, Π_f_, whereas the intermittency exponents are determined by the fluctuations of the energy flux (*46*). The polymers change the mean substantially, but the fluctuations are still dominated by the fluctuations of viscous energy dissipation which remains unchanged on the addition of polymers.

To check this hypothesis, we now use an alternative way to explore intermittency: through the statistics of the viscous dissipation. We find that the *q*th moment of the viscous dissipation averaged over a ball of radius ℓ shows scaling, viz.

$$\langle \epsilon_{\ell }^{q}\rangle ∼\ell^{\lambda_{q}},\;\mathrm{where}$$ (10A)

$$\epsilon_{\ell }\equiv \frac{2\mu_{f}}{\rho_{f}}{\left\langle S_{\alpha \beta }S_{\alpha \beta }\right\rangle }_{\ell }$$ (10B)

Here, the symbol ⟨·⟩_ℓ_ denotes averaging over a ball of radius ℓ and the symbol ⟨·⟩ averaging over the statistically stationary state of turbulence. For ℓ = *L*, ⟨ϵ*_L_*⟩ = ε_f_ the viscous dissipation in Eq. 2. The Legendre transform of the function λ_q_ gives the multifractal spectrum of turbulence *F*(α) which we plot in Fig. 5C. Our results, for the Newtonian case, agrees with the experiments in the Newtonian turbulence (*56*). We find that the multifractal spectrum is the same with or without polymers, thereby confirming our hypothesis.

Together, these evidences point toward the following scenario. For small enough viscosity and for small enough *k* (*k_inj_* < *k* < *k_d_*), the energy flux has two contributions—the advective flux and the polymeric flux. In the inertial range, the advective flux dominates. In the elastic range, the polymeric flux dominates. However, the intermittency exponents are determined not by the mean value of the flux but by its fluctuations. The fluctuations are determined by the fluctuations of the viscous dissipation, which remains the same in both polymeric and Newtonian turbulence.

## DISCUSSION

Our simulations reach the highest Re and De numbers reached so far in numerical simulations of homegeneous and isotropic turbulence of polymer solutions. Hence, by modern nomenclature, we may call this elasto-inertial turbulence (*26*, *57*), which is merely a renaming of the traditional field of polymeric turbulence. We find that the central role of the polymers is that the cascade of energy, which, in the absence of polymers, is determined by the advective nonlinearity, is now carried by both the advective nonlinearity and the polymer stress but at different scales. At large scales, the energy flux through scales is dominated by the advective nonlinearity, while the polymer stress plays a subdominant role—this is reversed at smaller scales. This gives rise to two scaling ranges, the classical Kolmogorov one and the new elastic one. We emphasize that the new scaling we find is a purely elastic effect; the advective nonlinearity plays a subdominant role in the range of scales where the elastic scaling is observed. A comparison of different models of polymeric fluids confirms that elasticity and not shear thinning is crucial to observe the elastic scaling range in the energy spectrum. Thus, elasto-inertial turbulence appears to be inertial turbulence at large scale and a new elastic behavior—different from elastic turbulence—at smaller scales that are still larger than viscous scales. The viscous effects may dominate over the elastic effects for small Re, thereby making the elastic range disappear. Furthermore, we establish that this elastic behavior is nonmonotonic in Deborah number. A simple qualitative explanation for this effect is that when De ≫ 1, polymers are not able to properly stretch due to their time scale being much larger than the largest time scale of the fluid, thus acting as a filter of the velocity fluctuations. However, our simulations with passive polymers show that this scenario is not true (polymer extension increases monotonically with De ). Thus the nonmonotonic behavior observed by us cannot be captured by a theory that treats polymers as passive objects.

At present, there are no theories that help us understand the elastic scaling. The theory by Bhattacharjee and Thirumalai (*12*, *13*) predicted a new power-law scaling in elastic range, with an exponent of ξ = 3; by contrast, we observe ξ ≈ 2.3, consistent with recent experiments (*33*). Bhattacharjee and Thirumalai also assumed that most of the polymers have not undergone coil-stretch transition. In our simulations, this may be true at small De , where the elastic range is nonexistent, but this is definitely not the case at high De where we do observe the elastic scaling. The theory by Fouxon and Lebedev (*14*) also predicts a power-law scaling (ξ > 3) and the existence of an elastic range, but we agree with Zhang *et al.* (*33*) that “the assumptions and quantitative prediction of the theory are not supported by” our numerics. We believe that the elastic range we observe is distinct from elastic turbulence in two ways. One, the scale-dependent Reynolds number in the elastic range is not necessarily very small. Two, we find ξ ≈ 2.3, whereas almost all studies of elastic turbulence find ξ > 3 (*18*, *23*, *35*, *36*, *58*, *59*), consistent with the theory of Fouxon and Lebedev (*14*). Note that at least one other simulation of elastic turbulence (*60*) has found ξ < 3 in two dimensional polymeric flows.

Our simulations extend the recent experiments by Zhang *et al.* (*33*), who did not probe the Deborah number dependence, by measuring quantities that are not easily accessible in the experiments, e.g., the contribution from the polymeric stress and the PDF of polymer extension, thereby providing constraints and clues to a future theory. We show that the polymer contribution can be decomposed into a purely dissipative term and into a purely energy flux, with the latter transporting the majority of energy in the elastic range. Its validity has been confirmed in several ways: (i) its span is consistent with the range of the elastic scale in the energy spectra; (ii) the polymer energy spectra exhibit a scaling consistent in range and slope with it. Last, we show that the intermittency corrections are the same in the elastic and the Newtonian cases. This indicates that the statistical nature of the fluctuations of the energy flux remains unchanged on addition of polymers—the fluctuations are determined by the statistics of the viscous energy dissipation, which remains the same in both polymeric and Newtonian turbulence.

## MATERIALS AND METHODS

The viscoelastic fluid is governed by the conservation of momentum and the incompressibility constraint

$$\rho_{f}\left(\frac{\partial u_{\alpha }}{\partial t}+\frac{\partial u_{\alpha }u_{\beta }}{\partial x_{\beta }}\right)=-\frac{\partial p}{\partial x_{\alpha }}+\frac{\partial }{\partial x_{\beta }}\left(2\mu_{f}S_{\alpha \beta }+\frac{\mu_{p}}{\tau_{p}}fC_{\alpha \beta }\right)$$ (11A)

$$\frac{\partial u_{\alpha }}{\partial x_{\alpha }}=0$$ (11B)

In the previous set of equations, ρ_f_ and μ_f_ are the density and dynamic viscosity of the fluid, *p* is the pressure, and S is the rate-of-strain tensor with components *S*_αβ_ defined as *S*_αβ_ = (*∂u*_α_/*∂x*_β_ + *∂u*_β_/*∂x*_α_)/2. The last term in the momentum equation is the non-Newtonian contribution, with μ_p_ being the polymer viscosity, τ_p_ the polymer relaxation time, *f* a scalar function, and C the conformation tensor with components *C*_αβ_ found by solving the following transport equation

$$\frac{\partial C_{\alpha \beta }}{\partial t}+u_{\gamma }\frac{\partial C_{\alpha \beta }}{\partial x_{\gamma }}=C_{\alpha \gamma }\frac{\partial u_{\beta }}{\partial x_{\gamma }}+C_{\gamma \beta }\frac{\partial u_{\gamma }}{\partial x_{\alpha }}-\frac{fC_{\alpha \beta }-\delta_{\alpha \beta }}{\tau_{p}}$$ (12)

The function *f* is equal to *f* = 1 in the purely elastic Oldroyd-B model and to *f* = (ℒ^2^ − 3)/(ℒ^2^ − *C*_γγ_) in the FENE-P model (ℒ is the maximum polymer extensibility) exhibiting both shear thinning and elasticity. Turbulence is sustained by an additional forcing in the momentum equation; in particular, we use the spectral scheme by Eswaran and Pope (*45*) to randomly injecting energy within a low–wave number shell with 1 ≤ *k* ≤ 2.

The equations of motion are solved numerically within a periodic cubic domain box of length 2π, discretized with *N* = 1024 grid points per side with a uniform spacing in all directions, resulting in a total number of around 1 billion grid points. The grid resolution *k*_max_ used in the present work is the largest used for viscoelastic fluids and is sufficient to represent all the relevant quantities of interest till the Kolmogorov length-scale η (*k*_max_η ≈ 1.7) (*49*). Furthermore, the smallest temporal scale of the flow, i.e., the Kolmogorv time scale τ_η_, is overly resolved (by two orders of magnitude τ_η_/Δ*t* ≈ 600), due to stability constraint arising from the non-Newtonian features of the flow, strongly increasing the computational cost. We have confirmed that these results are robust with respect to change in spatial and temporal resolutions, as reported in fig. S2 where the energy spectra obtained by different time and space resolutions are compared, finding the robustness of the reported results. To solve the problem, we use the flow solver Fujin, an in-house code, extensively validated and used in a variety of problems (*61*–*67*), based on the (second-order) finite difference method for the spatial discretization and the (second-order) Adams-Bashforth scheme for time marching. See also https://groups.oist.jp/cffu/code for a list of validations. The non-Newtonian stress equation is solved following the (exact) log-conformation approach (*68*) to ensure the positive definiteness of the tensor even at high De , without the addition of any artificial stabilising terms.
