# Supplementary material for: Large is different: Nonmonotonic behavior of elastic range scaling in polymeric turbulence at large Reynolds and Deborah numbers
Source: Sci Adv. 2023 Mar 15;9(11):eadd3831. doi: 10.1126/sciadv.add3831 (PMC10017036; doi:10.1126/sciadv.add3831)
Supplement: Supplementary file 1 — Figs. S1 to S4 Table S1 [file sciadv.add3831_sm.pdf]

Science Advances
AAAS

Supplementary Materials for

Large is different: Nonmonotonic behavior of elastic range scaling in polymeric turbulence at large Reynolds and Deborah numbers

Marco E. Rosti *et al.*

Corresponding author: Marco E. Rosti, marco.rosti@oist.jp

Sci. Adv. **9**, eadd3831 (2023)
DOI: 10.1126/sciadv.add3831

This PDF file includes:

Figs. S1 to S4
Table S1

SUPPLEMENTARY INFORMATION

Energy balance equation

We perform the Fourier transform of the governing equations to obtain an expression for the turbulent kinetic energy spectrum $\hat{E}(\boldsymbol{\kappa}, t) \equiv \frac{1}{2}\rho\langle\hat{\boldsymbol{u}}^* \cdot \hat{\boldsymbol{u}}\rangle$, where $\hat{(\cdot)}$ denotes the Fourier transform into the spectral space, $\boldsymbol{\kappa}$ denotes the wave vector with a magnitude κ, and the superscript $*$ denotes the complex conjugate;

$$\boldsymbol{\kappa} \cdot \hat{\boldsymbol{u}} = 0, \tag{S1}$$

$$\rho\frac{\mathrm{d}\hat{\boldsymbol{u}}}{\mathrm{d}t} + \hat{\boldsymbol{G}} = -i\boldsymbol{\kappa}\hat{p} - \mu_\mathrm{f}\kappa^2\hat{\boldsymbol{u}} + \hat{\boldsymbol{F}}_\mathrm{pol} + \hat{\boldsymbol{F}}, \tag{S2}$$

where $\hat{G}$ is the Fourier coefficient of the non-linear convective term appearing in the momentum equation, and i is the imaginary unit. Similar equations can be obtained for the complex conjugate $\hat{\boldsymbol{u}}^*$. When the momentum equation is multiplied by $\hat{\boldsymbol{u}}^*$, the pressure term $-i\boldsymbol{\kappa}\cdot\hat{\boldsymbol{u}}^*\hat{p}$ vanishes due to the incompressibility constraint, and the viscous term $-\mu_\mathrm{f}\kappa^2\hat{\boldsymbol{u}}\cdot\hat{\boldsymbol{u}}^*$ can be expressed in terms of the kinetic energy; $-2\mu_\mathrm{f}\kappa^2\hat{E}$. The same holds when multiplying the momentum equation of $\hat{\boldsymbol{u}}^*$ by $\hat{\boldsymbol{u}}$. By summing the two equations for $\hat{\boldsymbol{u}}$ and $\hat{\boldsymbol{u}}^*$ and dividing by 2, we have an expression for the time evolution of turbulent kinetic energy $\hat{E}(\boldsymbol{\kappa}, t)$

$$\frac{\mathrm{d}\hat{E}(\boldsymbol{\kappa})}{\mathrm{d}t} = \hat{T}(\boldsymbol{\kappa}) + \hat{V}(\boldsymbol{\kappa}) + \hat{F}_\mathrm{pol}(\boldsymbol{\kappa}) + \hat{F}(\boldsymbol{\kappa}), \tag{S3}$$

where the terms on the right-hand side represent the following contributions: $\hat{T} = -\frac{1}{2}(\hat{\boldsymbol{G}}\cdot\hat{\boldsymbol{u}}^* + \hat{\boldsymbol{G}}^*\cdot\hat{\boldsymbol{u}})$ is due to the non-linear convective term, $\hat{V} = -2\mu_\mathrm{f}\kappa^2\hat{E}$ is due to the fluid dissipation term, $\hat{F}_\mathrm{pol} = \frac{1}{2}(\hat{\boldsymbol{F}}_\mathrm{pol}\cdot\hat{\boldsymbol{u}}^* + \hat{\boldsymbol{F}}^*_\mathrm{pol}\cdot\hat{\boldsymbol{u}})$ is due to the non-Newtonian stress, and $\hat{F}_\mathrm{inj} = \frac{1}{2}(\hat{\boldsymbol{F}}_\mathrm{inj}\cdot\hat{\boldsymbol{u}}^* + \hat{\boldsymbol{F}}^*_\mathrm{inj}\cdot\hat{\boldsymbol{u}})$ is due to the external forcing. The one-dimensional energy spectrum $E(\kappa, t)$ can be obtained by isotropically averaging (S3) over the sphere of radius κ (i.e., $E(\kappa, t) = \iint_{S(\kappa)} \hat{E}(\boldsymbol{\kappa}, t)\mathrm{d}S(\kappa)$, where $S(\kappa)$ is the sphere defined by $\boldsymbol{\kappa}\cdot\boldsymbol{\kappa} = \kappa^2$),

$$\frac{\mathrm{d}E(\kappa)}{\mathrm{d}t} = T(\kappa) + V(\kappa) + F_\mathrm{pol}(\kappa) + F_\mathrm{inj}(\kappa). \tag{S4}$$

where the time derivative becomes zero for a statistically stationary flow. Integrating the equation above from κ to infinity, we obtain the energy-transfer balance

$$0 = \Pi_\mathrm{f} + \mathcal{D}'_\mathrm{f} + \mathcal{P}' + \mathcal{F}_\mathrm{inj}, \tag{S5}$$

where $\Pi_\mathrm{f}(\kappa) \equiv \int_\kappa^\infty T(\kappa)\,\mathrm{d}\kappa$, $\mathcal{D}'_\mathrm{f}(\kappa) \equiv \int_\kappa^\infty V(\kappa)\,\mathrm{d}\kappa$, $\mathcal{F}_\mathrm{inj}(\kappa) \equiv \int_\kappa^\infty F_\mathrm{inj}(\kappa)\,\mathrm{d}\kappa$, and $\mathcal{P}'(\kappa) \equiv \int_\kappa^\infty F_\mathrm{pol}(\kappa)\,\mathrm{d}\kappa$ represent the contributions to the spectral power balance from the non-linear convective, fluid dissipation, turbulence forcing, and non-Newtonian terms, respectively. The fluid dissipation term can be expressed as $\mathcal{D}_\mathrm{f}(\kappa) = -\int_0^\kappa V(\kappa)\,\mathrm{d}\kappa = \mathcal{D}'_\mathrm{f}(\kappa) + \langle\varepsilon_\mathrm{f}\rangle$, where $\langle\varepsilon_\mathrm{f}\rangle = -\int_0^\infty V(\kappa)\,\mathrm{d}\kappa$ is the rate of energy dissipated by the fluid viscosity. Similarly, the non-Newtonian contribution can be written as $\mathcal{P}(\kappa) = -\int_0^\kappa F_\mathrm{pol}(\kappa)\,\mathrm{d}\kappa = \mathcal{P}'(\kappa) + \langle\varepsilon_\mathrm{p}\rangle$, where $\langle\varepsilon_\mathrm{p}\rangle = -\int_0^\infty F_\mathrm{pol}(\kappa)\,\mathrm{d}\kappa$ is the non-Newtonian dissipation rate. Substituting these in the above equation, we obtain the energy balance equation used in the main document.

Total energy budget

The total energy of a FENE-P polymeric fluid system described by the governing equations is given by [17]:

$$F \equiv \underbrace{\frac{1}{2}\left\langle|\boldsymbol{u}|^2\right\rangle}_{\mathcal{E}_\mathrm{f}} + \underbrace{\frac{\mu_\mathrm{p}}{2\rho_\mathrm{f}\tau_\mathrm{p}}\left[\left\langle(L^2-3)\log(f)\right\rangle + 3\right]}_{\mathcal{E}_\mathrm{p}}, \tag{S6}$$

where $\mathcal{E}_\mathrm{f}$ is the kinetic energy, and $\mathcal{E}_\mathrm{p}$ is the contribution due to the polymer conformation. Note that by taking the limit $L \to \infty$ we get the corresponding expression for the Oldroyd-B model, $\mathcal{E}_\mathrm{p} = (\mu_\mathrm{p}/2\rho_\mathrm{f}\tau_\mathrm{p})\langle C_{\gamma\gamma}\rangle$. By taking the time-derivative of (S6), and using equations (1a), (1b) we get the following energy budget equation

$$\frac{\partial F}{\partial t} = \underbrace{-\frac{2\mu_\mathrm{f}}{\rho_f}\langle S_{\alpha\beta}S_{\alpha\beta}\rangle}_{\varepsilon_\mathrm{f}} \underbrace{-\frac{\mu_\mathrm{p}}{2\rho_\mathrm{f}\tau_\mathrm{p}^2}\langle f(fC_{\mu\mu}-3)\rangle}_{\varepsilon_\mathrm{p}}. \tag{S7}$$

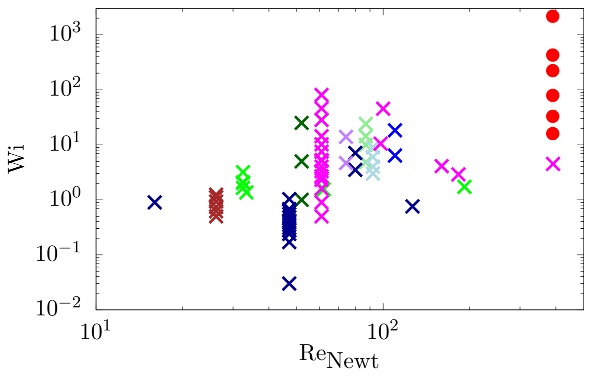

FIG. S1. **DNS data of polymeric turbulence available in the literature.** The plot reports the reference Reynolds numbers of the Newtonian cases Re_{Newt} and the Weissenberg numbers Wi of the simulations available in the literature: the cross symbols represents the previous investigations, while the filled circles the present one. Light green: Berti *et al.* [18]; green: Nguyen *et al.* [24]; dark-green: Watanabe and Gotoh [21, 23]; light blue: Fathali and Khoei [22]; blue: De Lillo *et al.* [20]; dark blue: Perlekar *et al.* [8, 10]; magenta: Valente *et al.* [25, 26]; purple: De Angelis *et al.* [17]; brown: Cai *et al.* [11]. The results of the present work focus on a high Re and Wi region never investigated before.

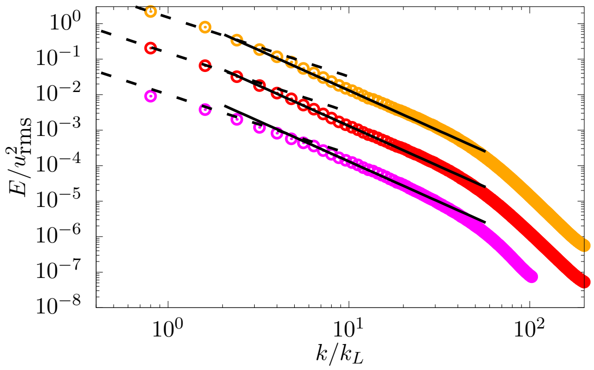

FIG. S2. **Numerical resolution.** Energy spectra for $De \approx 0.95$ obtained by (orange) reducing the timestep by a factor 10 and by (magenta) reducing the grid size by a factor 2. The spectra are shifted vertically for visual clarity. In both cases, the grid and time resolution proves to be appropriate.

Elasticity and shear-thinning

We consider three different models of polymeric fluids: the Oldroyd-B model (elasticity), the FENE-P model (elasticity and shear-thinning), and the Carreau–Yasuda model (shear-thinning), see Tab. S1 for the full list of simulations performed. In the inelastic shear-thinning fluid, the fluid viscosity μ_{f} is a function of the local shear rate $\dot{\gamma}$ as

$$\frac{\mu}{\mu_0} = \frac{\mu_\infty}{\mu_0} + \left(1 - \frac{\mu_\infty}{\mu_0}\right)\left[1 + (\tau_{\text{p}}\dot{\gamma})^2\right]^{\left(\frac{n-1}{2}\right)}, \tag{S8}$$

where μ_0 and μ_∞ are the viscosity at zero and infinite shear rates, n is the the power index ($n = 0.4$) and τ_{p} the consistency index. The model parameters are found to fit the FENE-P shear-thinning rheology, as shown in the inset of Fig. (S3A).

When comparing the results obtained for different non-Newtonian models, we find that the new power-law scaling in the energy spectra at intermediate small scales is a purely elastic effect, which completely disappear in the absence of elasticity while it is slightly reduced in range when shear-thinning is present together with elasticity, as shown in Fig. (S3A). When both elasticity and shear-thinning effects are present, the exponent of the power-law scaling remains practically unchanged if the parameter $\mathcal{L}$ (the maximum possible extension of the polymers) of the FENE-P model is varied within a reasonable range, while the elastic scaling range can completely disappear for too small values, showing a complex behaviour. While the presence of shear-thinning in combination with elasticity does not alter the

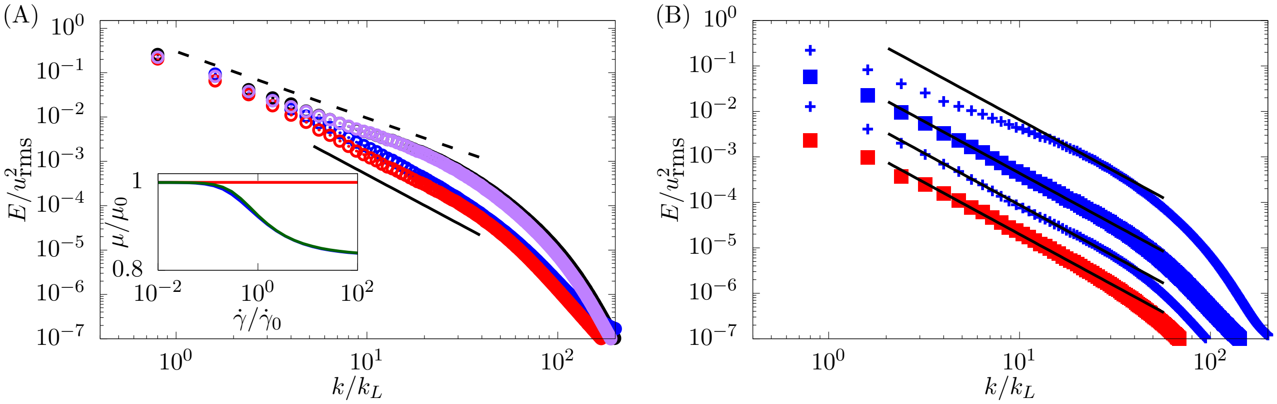

FIG. S3. **Effect of shear-thinning and elasticity on the energy spectra.** (A): Energy spectra for $\mathrm{De} \approx 0.95$ for different fluids. Black: Newtonian fluid. Purple: inelastic Carreau–Yasuda model. Blue: FENE-P model. Red: Oldroyd-B model. The inset shows the shear rheology of the same fluids. The new scaling at intermediate small scales is a purely elastic effect, which completely disappear in the absence of elasticity while it is reduced when shear-thinning is present together with elasticity. (B): Energy spectra for $\mathrm{De} \approx 0.95$ for FENE-P fluids with different $\mathcal{L}$. From top to bottom: $\mathcal{L} = 20$, 60, 100, ∞ (Oldroyd-B model). The spectra in the right panel are shifted vertically for visual clarity. The new scaling at intermediate small scales is present for all cases with sufficiently large $\mathcal{L}$. The dashed and solid lines represent the scaling $k^{-\psi}$ with $\psi = 5/3$ and $\psi \approx 1.35$.

slope of the elastic range, it reduces its range: this is caused by an enhancement of the non-linear energy flux Π_f and a consequent reduction of the polymer flux, Π_p, as shown in Fig. (S4A). In the FENE-P model the extension of the polymers is arrested due to two mechanisms. One because of the feedback to the flow, and two because of the nonlinear saturation term in the FENE-P equation itself. If $\mathcal{L}$ is small, the nonlinear saturation term stops the polymers from having large extensions. This implies that the feedback from the polymer to the flow is also small, consequently the flux Π_p is also small. Hence, as $\mathcal{L}$ is increased, Π_p increases and Π_f decreases. When $\mathcal{L} \to \infty$, which corresponds to the Oldroyd-B model, we get the largest possible values for Π_p. In this case the elastic range is best observed.

TABLE S1. List of all cases considered in the present work.

case	model	Re_λ	De	Wi	$\mu_\mathrm{p}/(\mu_\mathrm{p}+\mu_\mathrm{f})$	$\mathcal{L}$
○	single phase	390	–	–	0.1	–
⬠	Oldroyd-B	480	0.18	16	0.1	∞
◊	Oldroyd-B	630	0.37	33	0.1	∞
○	Oldroyd-B	740	0.95	79	0.1	∞
△	Oldroyd-B	690	2.31	223	0.1	∞
▽	Oldroyd-B	614	4.82	427	0.1	∞
□	Oldroyd-B	447	24.6	2169	0.1	∞
+	FENE-P	420	0.95	79	0.1	20
○	FENE-P	610	0.95	79	0.1	60
×	FENE-P	700	0.95	79	0.1	100
○	FENE-P	620	2.31	31	0.1	60
○	FENE-P	487	4.82	31	0.1	60
○	Carreau–Yasuda	370	–	–	–	–

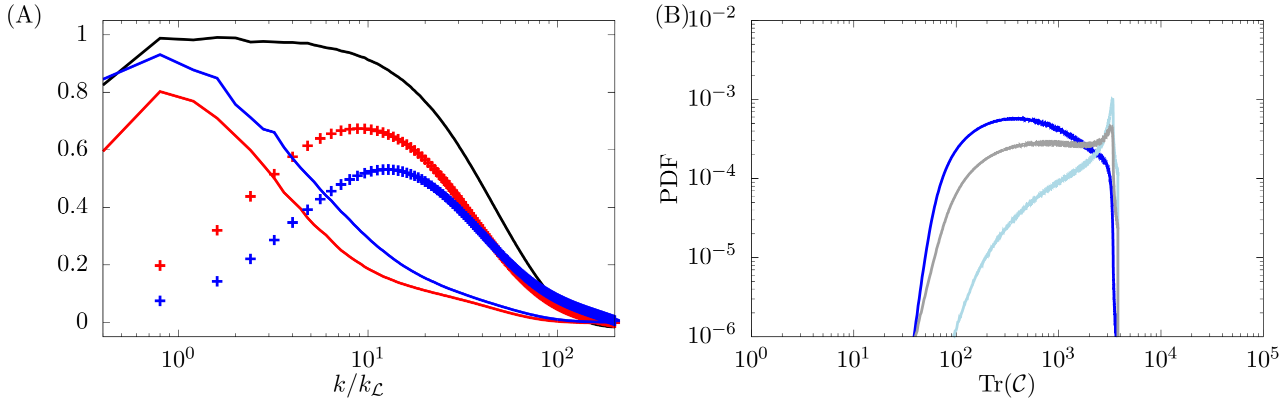

FIG. S4. **Effect of shear-thinning on the energy balance and polymer extension.** (A): the non-linear energy flux Π_f (solid line) and polymer flux, Π_p (+) for the (black) Newtonian, (red) Oldroyd-B and (blue) FENE-P fluids at $De \approx 1$. In the FENE-P fluid, the non-linear energy flux is stronger than in the Oldroyd-B case, resulting in a reduced polymer flux. (B): Probability distribution function (PDF) of the polymer extension for different Deborah numbers, measured in terms of $\mathrm{Tr}(\mathcal{C})$, obtained with the FENE-P model. Blue: De ≈ 0.95; light blue: De ≈ 2.31; gray: De ≈ 4.82. Differently from the results obtained with the Oldroyd-B model, the polymer extension is upper bounded by $\mathcal{L}^2$; however, the non-monotonic trend with De is still evident.
